# Investigation of the Fracture and Fragmentation of Implosively Driven Thin-Walled Cylindrical Shell: From Thermodynamic Analysis to CDEM Simulation

**DOI:** 10.3390/ma16165619

**Published:** 2023-08-14

**Authors:** Yinzhe Ou, Jianfei Yuan, Qindong Lin, Wenjun Jiao, Junming Yuan, Jianjun Su, Chun Feng, Xinghan Li, Yundan Gan

**Affiliations:** 1 Xi’an Modern Chemistry Research Institute, Xi’an 710065, China; yinzhe_ou@163.com (Y.O.);; 2 School of Environment and Safety Engineering, North University of China, Taiyuan 030051, China; 3 School of Engineering Science, University of Chinese Academy of Sciences, Beijing 100190, China; 4 School of Physical Science and Technology, Southwest Jiaotong University, Chengdu 610031, China

**Keywords:** shelled charge, equation of state, evolution of cracks, fragments scattering

## Abstract

The scattering of fragments is a notable characteristic of the explosive detonation of a shelled charge. This study examines the fracture and fragmentation of the shell and the process by which natural fragments form under the strains of implosion. The analysis takes into account both the explosive’s energy output and the casing’s dynamic response. For this purpose, utilizing a thermochemical code as an alternative to the conventionally employed cylinder test, the Jones–Wilkins–Lee equation of state (JWL EOS) was calibrated within a range of relative specific volume up to 13. The detonation of the shelled charge was subsequently analyzed using the continuum–discontinuum element method (CDEM). Following this, the formation mechanisms and scattering characteristics of natural fragments were scrutinized. The analysis found that the shell predominantly experiences shear failure with uniform evolution, displaying a “hysteresis effect” and two mutation stages in the evolution of tensile failure. Within the JWL EOS’s calibrated range, the representation of fragment displacement and velocity improved by 47.97% and 5.30%, respectively. This study provides valuable guidance for designing the power field of warheads and assessing their destructive power.

## 1. Introduction

Fragments generated by shelled charge explosions exhibit certain attributes, namely, rapid speed, minute size, large quantity and high destructive force. To forecast uncontrolled warhead detonations and aid in the design of controlled warheads, it is imperative to research the fracture laws and scattering characteristics of shells [1,2,3].

The velocity and mass–quantity distribution of natural fragments, which are principal measures of destructive potency, are intrinsically linked to the energy output of explosives and the dynamic mechanical characteristics of the shell [4,5,6]. The equation of state for detonation products delineates the former, while a shell’s material and structural parameters set the latter. The interaction of these factors determines how natural fragments form and scatter.

The equation of state, which depicts the relationships between the parameters of detonation products under the thermodynamic equilibrium state, is a crucial representation of an explosive’s work capacity. Numerous forms of the equation of state have been developed. The semi-empirical equations of state, such as JWL EOS, which do not consider product composition change, are simple yet dependably accurate within certain temperature and pressure ranges, making them widely used in finite element calculation programs [7]. However, their application is constrained because of over-simplification of the detonation problem from a phenomenological perspective. Thermochemical equations of state such as BKW [8], WCA [9], and VLW [10] consider the chemical reaction process and composition change. Although such equations of state are widely used in basic scientific calculations, their computational intensity impedes their application in finite or discrete element programs aiming to characterize explosive work capacity [11,12,13,14,15]. Thus, by calibrating coefficients of semi-empirical equations of state via the chemical reaction perspective both computational efficiency and reliability can be taken into account.

Under implosion load, the shell undergoes plastic deformation, crack proliferation and dynamic fragmentation [16,17,18]. Such processes have been extensively studied through recent experiments and numerical simulations. Based on physically-based material constitutive and failure algorithms in the Eulerian numerical framework, Cullis et al. [19] analyzed the fracture of thick-walled EN24 steel cylinders filled with PBXN-109 explosive. To optimize the description of the dimensions with the support of experimental data, Felix et al. [20] developed a rapid generation model for the shape and size of blast fragments. To analyze the dispersion of prefabricated fragments in the full spatial and temporal domain, Wang et al. [21] used the continuum–discontinuum element method [22] to investigate the large deformation and cross-scale calculations of the warhead rupture process. The current research on shell fracture and fragments formation additionally includes different shell thicknesses [23], variable charge geometries [24], ductile metal shear fracture [25,26], end-constrained casing fracture [27], cylindrical discontinuous charges [28], axial velocity distribution [29,30,31], and the effect of initial velocity on fragment size [32,33]. Currently, natural fragment formation studies mainly involve thick-walled shells and low charge-to-shell ratios, resulting in slower initial fragment scattering velocities [34,35,36].

Despite a great deal of research around natural fragments, two issues persist. First, the calibration of the JWL EOS coefficients is limited by the cylinder test. As such, it is challenging to depict a broader temperature and pressure range of the explosive’s work capacity, while the shell demand exceeds the range offered by cylinder test. Second, using the finite element method to study fracture issues hardly ensures mass conservation, making accurate depiction of both shell fracture mechanism and fragment scatter characteristics difficult to achieve. In particular, thin-walled shell rupture produces smaller mass and faster fragments, which is a less explored trait in the study of shell fracture and fragment velocity characteristics.

This study delves into the shell fracture and fragment scattering of thin-walled cylindrical shelled charges based on the thermochemical equation of state and continuum–discontinuum element method. Initially, the work capacity of mixed explosive HMX/RDX (70%/30%) was characterized by considering the chemical reaction process of detonation products. The isentropic expansion curve of the product was computed by the thermochemical code DLCHEQ. Coefficients of the JWL EOS were calibrated through curve fitting and subsequently validated via cylinder test. Following this, a shelled charge explosion test was designed and simulated using the continuum–discontinuum element method. Substituting the coefficients into the CDEM program, the process of the shell and ensuing acceleration of the fragments was simulated from fracture to fragmentation. The reliability of the simulation results was verified by the static explosion velocimetry test and Mott theory, and the fracture mechanism of the shell and acceleration evolution of fragments along the axial direction was further explored.

## 2. Thermodynamic State of Detonation Products

To explore the work capacity of explosives across a wider temperature and pressure ranges, the thermochemical attributes of the detonation products should be analyzed and their thermodynamical characteristics characterized. Compositional evolution and isentropic expansion process of detonation products were ascertained utilizing the thermochemical equation of state and the classical CJ detonation model. The JWL EOS coefficients were calibrated by fitting the isentropic expansion curve, which was subsequently validated for reliability through the cylindrical test.

### 2.1. Calculation of Chemical Reaction Perspective

The work capacity of explosives is substantially influenced by the thermodynamic parameters of detonation products such as pressure and temperature. Therefore, a comprehensive evaluation of explosive performance requires further procurement of CJ detonation parameters such as detonation velocity, pressure, temperature, and entropy. Using the DLCHEQ, the CJ point state parameters of the explosives were computed. Thereafter, the isentropic pressure–volume (P-V) relationship of the detonation products was calculated by leveraging the values of the entropy and detonation pressure.

#### 2.1.1. Thermochemical Code

**Figure 1:**
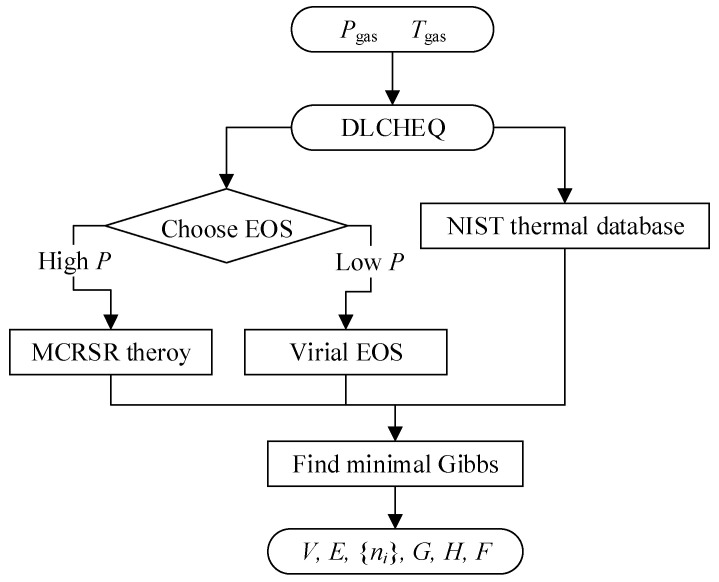
Schematic diagram of EOS of detonation products using DLCHEQ.

DLCHEQ is a thermochemical code developed based on the fundamental principles of thermodynamics and thermochemistry [37,38]. It demonstrates reliable precision in describing the equation of state of detonation products over a wide spectrum of temperature and pressure. This capability allows for the comprehensive characterization of detonation products spanning from tens of GPa to atmospheric pressure and from thousands of K to room temperature. DLCHEQ can be divided into three core sections: the unreacted explosive equation of state, the EOS of product, and the model for reaction rate. According to different pressure ranges, the MCR equation (applicable from tens of GPa to hundreds of MPa), the second-order Virial equation (applicable from hundreds of MPa to atmospheric pressure), and the ideal gas equation (applicable from below atmospheric pressure) are applied in DLCHEQ. The EOS of detonation products encompasses sixteen distinct components, including CO_2_, O_2_, H_2_, H_2_O, CH_4_, NH_3_, NO, N_2_, CO, N, O, N_2_O, NO_2_, C, Al, and Al_2_O_3_. Through the input parameters of the explosive’s molecular formula, the molecular formula of each detonation product component, and the explosive’s initial density, DLCHEQ effectively generates the essential thermodynamic quantities of the detonation products, including volume, internal energy, composition, and enthalpy. Depending on the pressure, different equations of state are chosen to describe different gas components. After the temperature, pressure, and constituent components are identified, all the thermodynamic parameters of the detonation product system can be calculated through mass weighting. The sequence of these calculations is encapsulated in Figure 1.

In assessing the states of chemical equilibrium and related calculations, the Gibbs free energy reaches its minimum value amid the chemical equilibrium condition. The chemical equilibrium equations are introduced as follows:

(1) $$\{\begin{array}{ll} \frac{n_{i}}{n_{i}^{0}}-\frac{n}{n^{0}}+\sum_{k=1}^{c}\lambda_{k}\alpha_{ik}=-f_{i}\left(n^{0}\right) & i=1,\ldots ,s\\ \sum_{k=1}^{c}\lambda_{k}\alpha_{ik}=-G_{i}\left(n^{0}\right) & i=s+1,\ldots ,t\\ \sum_{i=1}^{t}\alpha_{ij}n_{i}=b_{j} & j=1,\ldots ,c \end{array}$$

where *α_ik_* denotes the number of atoms of the kth element in the molecular formula of the ith component, *λ_k_* denotes the chemical potential of the ith element, *n_i_* and *n*0 denote the numbers of moles before and after the iteration of the ith kind, respectively, and *n* and *n*_0_ denote the total number of moles before and after the iteration of the gas phase product, respectively. The calculation formulas are as follows:$n=\sum_{i=1}^{s}n_{i}$, $n^{0}=\sum_{i=1}^{s}n_{i}^{0}$, where *f_i_(n*^0^*)* denotes the free energy of the ith kind of gas phase product and *G_i_(n*^0^*)* denotes the free energy of the ith kind of solid phase product.

Through DLCHEQ, the isentropic expansion process of detonation products from the CJ state to the state of relative specific volume $\bar{v}$ = 13 were computed, along with the compositional evolution of detonation products and the isentropic expansion line.

This article focuses on a mixed explosive comprising 70% HMX (C_4_H_8_N_8_O_8_) and 30% RDX (C_3_H_6_N_6_O_6_) as the subject of calculation. The enthalpies of formation for the individual explosives are 0.255 kJ/g and 0.277 kJ/g, respectively, while their respective molecular weights are 296.155 and 222.116.

#### 2.1.2. Compositional Evolution

**Figure 2:**
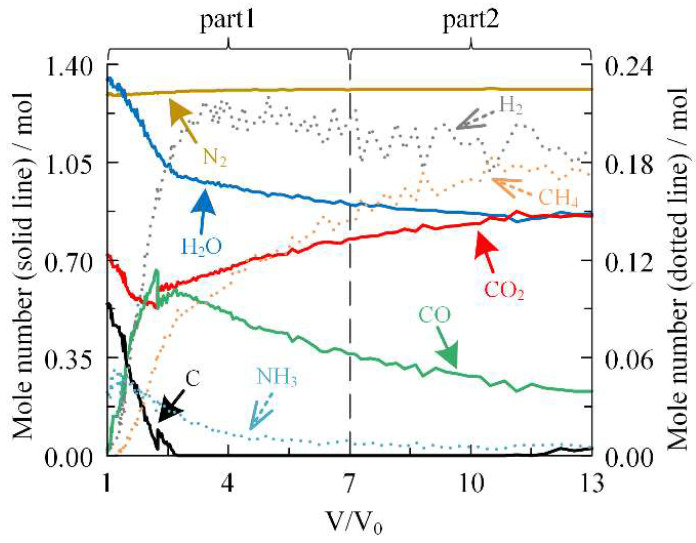
Compositional evolution of detonation products (70% HMX and 30% RDX).

Spanning the range of relative specific volume from 1 to 13, the compositional evolution of detonation products is illustrated in Figure 2. Due to the order of magnitude difference of content, constituent components were represented by solid and dotted lines. As can be seen, the system is predominantly composed of N_2_, H_2_O, CO_2_, and CO, with lesser quantities of NH_3_, CH_4_, and H_2_. The concentration of all component parts fluctuates noticeably, with the exception of N_2_. The H_2_O content diminishes consistently during expansion, while C and CO_2_ react to yield CO. When the C (graphite) concentration diminishes to zero, the increase in CO transitions to a decrease simultaneously with an increase in CO_2_. This suggests that the reaction experiences an initial exothermic process followed by heat absorption.

The constituent components undergo an overall evolution characterized by an initial rapid progression followed by deceleration. The evolution process of the constituent components is divided into two distinct parts demarcated by the measurable limits of the 25 mm cylinder test ($\bar{v}$ = 7). The first part reveals the occurrence of the primary reaction, while the reaction in the second part continues at a reduced rate. Comparing the end points of part 2 with part 1, the percentage of CO is 62.48% of the corresponding value, while CO_2_ amounts to 111.20% and CH4 amounts to 122.97% of their respective values in part 1. These findings illustrate the persistence of the reaction between detonation products beyond the measurable range of the cylinder test, exerting an influence on the compositional evolution.

The work capacity of explosives is influenced by the compositional evolution of the detonation products. However, in the process of formulating the empirical equation of state, the constituent compositions are usually regarded as unchanged. The range of measurement in the cylinder test is restricted by the tube breaks. As shown in Figure 2, the constituent components continue to change substantially during the evolution process after $\bar{v}$ = 7, directly affecting the work capacity of the explosives due to endothermic and exothermic reactions. Hence, opting for the thermochemical code is a better choice for calibrating the JWL EOS as opposed to relying on the cylinder test. This approach affords extended ranges of temperature and pressure for the expansion data of detonation products, leading to a more precise depiction of the work capacity of explosives.

#### 2.1.3. Isentropic Expansion Curve

The working range of detonation products is assumed as isentropic expansion. According to the computed entropy and detonation pressure, the isentropic P-V relationship of detonation products can be computed by DLCHEQ.

The shock compression state of the detonation products satisfies the equation of state; then, the Hugoniot relation is introduced as follows:

(2) $$\{\begin{array}{l} P=\frac{1}{N}\rho^{2}\frac{\partial F}{\partial \rho }\\ E=-T^{2}\frac{\partial }{\partial T}\left(\frac{F}{T}\right)\\ E_{H}-E_{0}=\left(P_{H}+P_{0}\right)\left(V_{0}-V_{H}\right)/2 \end{array}$$

where *ρ* denotes the particle number density. The pressure *P* and internal energy *E* of the system are obtained from the total free energy *F*, while *E*, *P*, and *V* are indicated by the subscripts 0 and *H* for the molar internal energy, pressure, and molar volume of the initial and shock-compressed final states of the system, respectively.

Because the detonation velocity of the CJ state is at the minimum, the CJ detonation velocity can be solved by the parabolic minimum method for the three points on the Hugoniot curve. The calculation formula is introduced as follows:

(3) $$D=V_{0}{\left(\frac{P_{H}-P_{0}}{V_{0}-V_{H}}\right)}^{1/2}$$

The main constituent components of the test charge in this paper are 70% HMX and 30% RDX. The CJ detonation parameters calculated by the above equation are shown as follows: detonation velocity *D_CJ_* = 9.1772 km/s, specific volume ${\bar{v}}_{CJ}$ = 0.4164 cm^3^/g, detonation temperature *T_CJ_* = 3818.2 K, detonation pressure *P_CJ_* = 35.286 MPa, internal energy *E_CJ_* = 2.266 kJ/g, entropy *S* = 7.3016 J/g/K. In this way, the isentropic expansion curve of detonation products can be obtained.

### 2.2. Calibration of JWL Equation of State

The coefficients were calibrated using JWL EOS to fit the isentropic curve computed through DLCHEQ. The two-dimensional fluid dynamics software DYNA-2D was employed to simulate the cylinder test to obtain the expansion curve of the external wall. The reliability of the coefficients derived from the calibration was validated through comparison with the results of the explosive cylinder test in the range of $\bar{v}$ < 7.

#### 2.2.1. Curve Fitting

**Figure 3:**
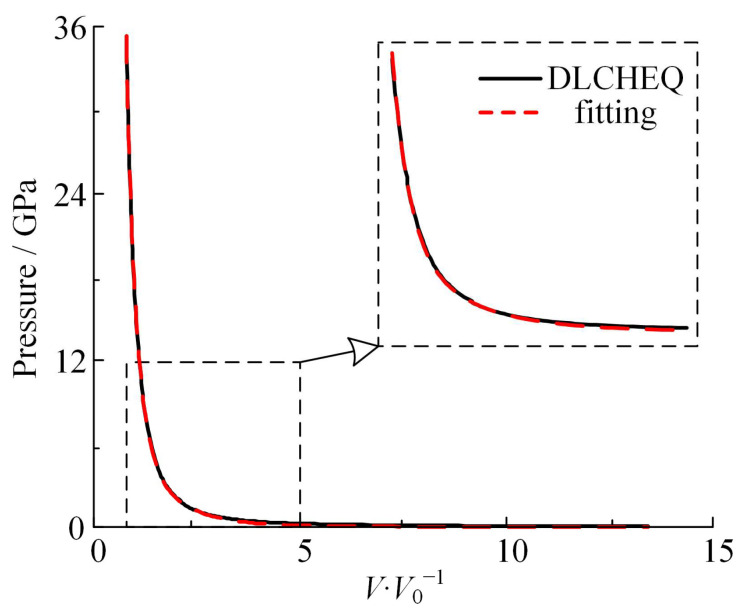
Using the JWL EOS fitting isentropic curve computed by DLCHEQ.

The fitting function $P=Ae^{-R_{1}\bar{v}}+Be^{-R_{2}\bar{v}}+C{\bar{v}}^{-(w+1)}$ is the form of the isentropic JWL EOS, where *P* denotes the instantaneous pressure of the detonation product. Only three of the six coefficients *A*, *B*, *C*, *R*_1_, *R*_2_, and *ω* to be calibrated are independent. A genetic algorithm was used to fit *R*_1_, *R*_2_, and *ω,* while *A*, *B*, and *C* were solved simultaneously according to three compatibility equations. The compatibility equation is established by the CJ condition, Hugoniot relation, and $-\left(\partial p_{s}/\partial V\right){\bar{v}}_{\mathrm{CJ}}=\rho_{0}D^{2}$ of the ideal detonation state, which are introduced as follows:

(4) $$\{\begin{array}{l} Ae^{-R_{1}{\bar{v}}_{CJ}}+Be^{-R_{2}{\bar{v}}_{CJ}}+C{\bar{v}}_{CJ}^{-(\omega +1)}=p_{CJ}\\ \frac{A}{R_{1}}e^{-R_{1}{\bar{v}}_{CJ}}+\frac{B}{R_{2}}e^{-R_{2}{\bar{v}}_{CJ}}+\frac{C{\bar{v}}_{CJ}^{-\omega }}{\omega }=E_{0}+\frac{1}{2}p_{CJ}\left(1-{\bar{v}}_{CJ}\right)\\ AR_{1}e^{-R_{1}{\bar{v}}_{CJ}}+BR_{2}e^{-R_{2}{\bar{v}}_{CJ}}+C(\omega +1){\bar{v}}_{CJ}^{-(\omega +2)}=\rho_{0}D_{CJ}^{2} \end{array}$$

where the initial internal energy *E*_0_ =10 GPa, the range of values of *R*_1_, *R*_2_, and *ω* is *R*_1_ = 4–5, *R*_2_ = 1–2, and *ω* = 0.2–0.4, respectively. The fitting results are shown in Figure 3. The maximum error on the isentropic curve Δ*P* = 0.6 GPa. A closer inspection of the curve’s intermediate section reveals a satisfactory fitting, suggesting that the genetic algorithm applied in this study accurately fits the product’s isentropic expansion curve.

Coefficients of the JWL EOS obtained from the calibration are shown in Table 1.

#### 2.2.2. Test Verification

**Figure 4:**
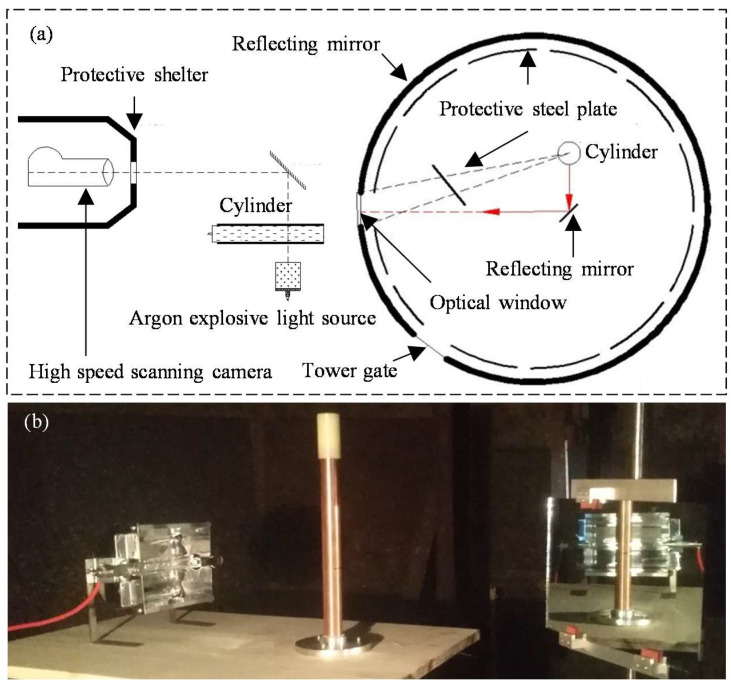
Configuration of cylinder test in explosive tower: (**a**) global view and (**b**) physical view.

A 25 mm cylinder test was conducted in the explosion tower, utilizing a xenon lamp for lighting purposes. Data were procured from an ultra-high-speed scanning camera operating at a velocity of 3 mm/μs and subsequently calibrated with the assistance of PDV laser interferometry. The designated observational vantage point was positioned 120 mm distant from the cylinder’s terminus. The sample, instrument and tower layout are shown in Figure 4. The test cylinder, composed entirely of TU1 oxygen-free pure copper (density of 8.93 g/cm^3^), exhibited a charge density of 1.86 g/cm^3^.

**Figure 5:**
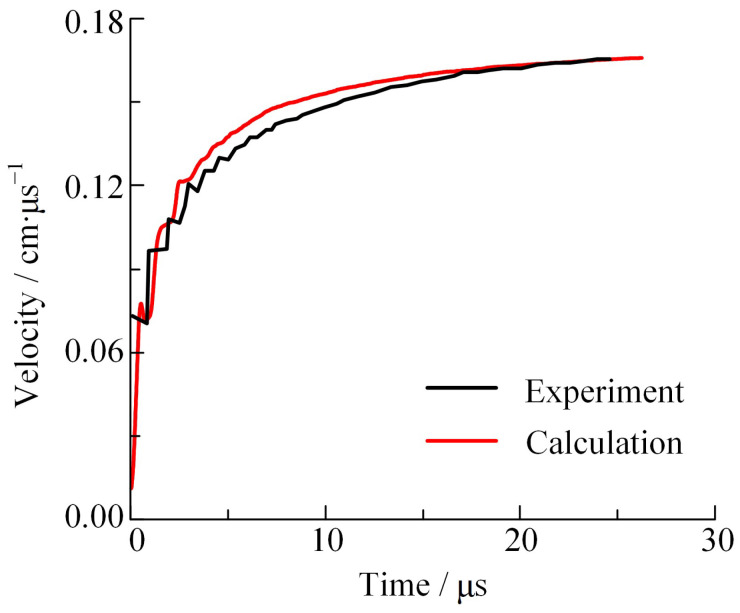
Results of the cylinder wall expansion curve test and calculation.

DYNA-2D two-dimensional fluid dynamics software was utilized to simulate the cylinder test. Both the Johnson–Cook constitutive model and the Gruneisen equation of state [39] were applied for the copper tube. The simulation results are presented in Figure 5, illustrating the expansion curve of the cylinder wall. The JWL EOS calibrated by the isentropic curve shows significant consistency with the test measurements, which can effectively describe the expansion process of the external wall in the cylinder test. Thus, the reliability of the JWL EOS, as calibrated in this study is substantiated, asserting its capability to accurately characterize the work capacity of explosives.

The accuracy of the JWL EOS was verified over the range of relatively specific volumes measured in the cylinder test. The 25 mm cylinder test can describe the work capacity of the explosive in the range of $\bar{v}$ < 7, while the explosive continues to exert a propulsive acceleration effect on fragments in $\bar{v}$ > 7. The thermochemical code DLCHEQ computes the isentropic expansion curve over a wider temperature–pressure range. This process takes into account both the composition of the explosive and the chemical reaction process. Consequently, the JWL EOS can characterize the explosive’s work capacity over a longer time interval.

## 3. Thin-Walled Cylindrical Shells Driven by Detonation Products

Driven by detonation products, the thin-walled shell fractures and natural fragments are subsequently produced. Experiments and corresponding numerical simulation were conducted to investigate the fracture characteristics of shells and acceleration of fragments. The velocities of the fragments were measured in the experiment, and the JWL EOS of detonation products obtained from the previous section was applied to the numerical simulation utilizing CDEM. Simulation results of the initial velocities and mass–quantity distribution of the fragments were validated with experimental results and Mott theory.

### 3.1. Experiment of Shelled Charge Explosion

**Figure 6:**
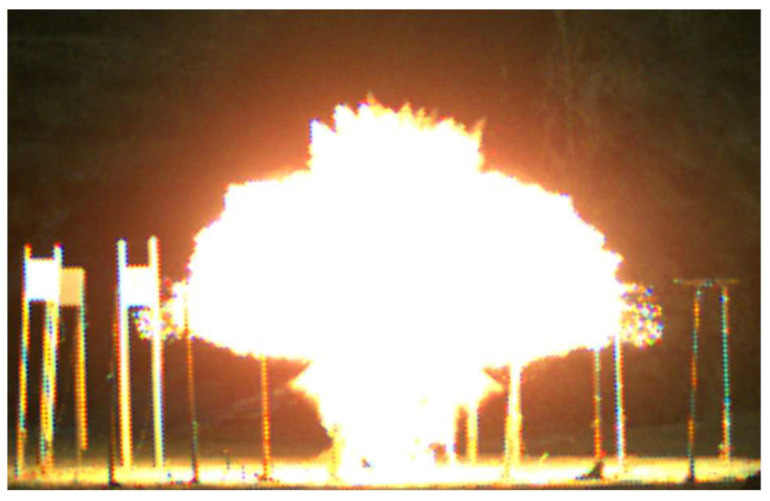
Explosion test of shelled charge.

A shelled charge explosion test was conducted to investigate the velocity and mass distribution of fragments. A Q235 steel shell with the following specifications was used in the experiment: length *L* = 285 mm, inner diameter *D_I_* = 84.2 mm, outer diameter *D_O_* = 88.8 mm, shell wall thickness *δ* = 2.3 mm, shell mass *M_S_* = 1.36 kg, charge mass *M_E_* = 3 kg. Fragment velocity was measured electrically with a comb-shaped target. There is a short-circuit in the velocity signal output when the fragment does not reach the target; conversely, the signal output is an open circuit when the fragment arrives at the target. This system offers accurate measurement of fragment velocities at various spatial locations, thereby mitigating the constraints of the traditional single-point through-break type velocity target. The impact of explosion and the scattering effect of fragments are illustrated in Figure 6. The energy release framework in a shelled charge explosion encompasses shockwaves, fragment dissemination, high-temperature fireballs, etc.

The velocity tests were executed at distances of 4 m and 5.2 m, forming three distinct test groups, with the collected data illustrated in Table 2. $V_{x}=V_{o}\times e^{-ax}$ is utilized for calculating the initial velocity of the fragment, where *V_x_* denotes the velocity of the fragment when it is scattered by *x* m, *V*_0_ denotes the initial speed of the fragment, and *a* denotes the coefficient to be fitted. Substituting the data in Table 2 gives *V*_0_ = 2.22 km/s. The comb target, being a type of on–off velocity measuring system, measures the velocity of the fragment that first impacts the target surface. Thus, under the static explosion condition of this experiment, the maximum velocity of the fragment is 2.22 km/s. The ratio of charge to shell mass for the test, *M_E_*/*M_S_* = 2.21, is typical of thin-walled shells, implying a high number of lighter, smaller fragments produced by the explosion. Considering the limitation of the recovery method, only a few parts of fragments were able to be recovered, with each weighing less than 3 g. These fragments account for a total mass less than that of the original shell, rendering it insubstantial as a comparative value.

### 3.2. Numerical Simulation Using Continuum–Discontinuum Element Method

In the midst of detonation product expansion, the metallic shell undergoes three stages, respectively, deformation, micro-crack initiation, and expansion, resulting in formation of natural fragments. To accurately portray the process of crack initiation and propagation while maintaining the consistency of momentum, energy, and mass, the continuum–discontinuum element method was employed in this study to investigate the fracture and fragmentation characteristics of the metal shell subjected to implosion loading.

#### 3.2.1. Basic Concept

The continuum–discontinuum element method (CDEM) can be defined as a dynamic explicit solution algorithm that functions within a Lagrangian framework. Strict control equations are established via the Lagrangian energy system, which, when combined with the dynamic relaxation method, allows for explicit iterative solutions. This enables the attainment of a unified depiction of continuum–discontinuum scenarios. In order to analyze material failure progression, the model takes into account fractures that occur both inside and on the boundaries of the block. The algorithm simulates the entire course, from continuous deformation of the material to eventual fracture and subsequent movement. This method integrates the benefits of both continuous and discontinuous calculation: the former implements techniques such as finite element, finite volume, and spring element, while the latter utilizes the discontinuous element method.

**Figure 7:**
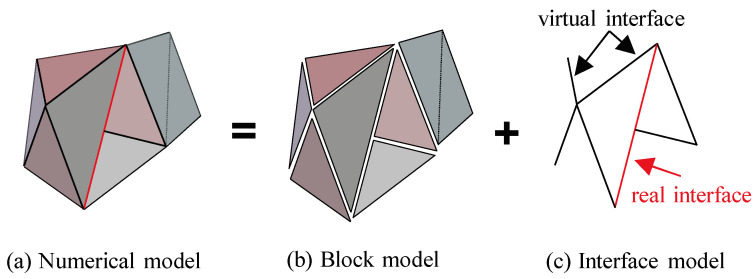
Schematic diagram of CDEM.

The numerical model in CDEM consists of two parts: block and interface. The block is composed of one or more finite element cells, which are used to characterize the continuous characteristics of the material such as elasticity, plasticity, and damage. The common boundary between the two blocks is the interface, which is used to characterize the discontinuous characteristics of the material such as fracture, slip, and collision. Within the CDEM framework, the interface includes two concepts: real interface and virtual interface. The real interface is used to characterize the real discontinuous surface, including the interface between constituent components. In contrast, the virtual interface serves dual functions. First, it connects two blocks to transmit mechanical information; second, it provides a potential channel for the propagation of explicit cracks (i.e., cracks can propagate along any virtual interface). The schematic diagram of the numerical model in CDEM is shown in Figure 7. The schematic model consists of seven blocks, of which one block is composed of two triangular elements and the remaining six blocks are composed of one triangular element each. In addition, the red line in Figure 7c is the real interface, while the black line is the virtual interface.

#### 3.2.2. Explosion Model

The model applies the standard form of the JWL EOS to delineate the expansion process of detonation products. The calculation formula is introduced as follows:

(5) $$P=A\left(1-\frac{\omega }{R_{1}\bar{v}}\right)e^{-R_{1}\bar{v}}+B\left(1-\frac{\omega }{R_{2}\bar{v}}\right)e^{-R_{2}\bar{v}}+\frac{\omega E_{0}}{\bar{v}}$$

where *E*_0_ denotes the specific internal energy of detonation products at the initial moment (J/m^3^). The values of *A*, *B*, *R*_1_, *R*_2_, and *ω* are shown in Table 1.

It is assumed that the ignition time of an explosive (including several units) is *t*_0_, the coordinates of the ignition point are (*x*_0_, *y*_0_, *z*_0_), the distance from the centre of a unit in the explosive to the ignition point is *d*, and the detonation velocity of the explosive is *D*, making the ignition time of the unit *t*_1_ = *d*/*D* + *t*_0_. When the explosion time *t* > *t*_1_, the explosion pressure is calculated according to Equation (6), where *P_r_* denotes the real explosion pressure; here, *P*(*v*, *E*) denotes the equation of state of detonation products based on the JWL model and *ξ* denotes the energy release rate, which can be obtained by Equation (7), where *V_e_* denotes the initial volume of the unit and *A_e_*_-max_ denotes the maximum area of the unit.

(6) $$P_{r}=\xi P\left(v,E\right)$$

(7) $$\xi =\{\begin{array}{ll} \min\left(\frac{2\left(t-t_{1}\right)DA_{e-\max}}{3V_{e}},1\right) & \left(t>t_{1}\right)\\ 0 & \left(t\leq t_{1}\right) \end{array}$$

Furthermore, this study presents a model for detonation product leakage, with the aim of depicting the decrease in pressure following shell break. Considering the inevitable dissipation of detonation gas post-explosion, an exponential decay function serves as an effective tool to articulate the decline in detonation gas pressure resulting from such dissipation. The calculation formula is introduced as follows:

(8) $$p_{r}=pe^{-{\left(t/t_{c}\right)}^{n}}$$

where *p* denotes the explosion pressure calculated according to the formula of the explosion source, *t_c_* denotes the characteristic time (when *t* = *t_c_*, the pressure becomes 36.8% of the original pressure.), *n* denotes the characteristic index, and *t_c_* is closely related to the geometry of the shell and the charge, which is chosen to be *t_c_* = 90 μs.

#### 3.2.3. Constitutive Model

**Figure 8:**
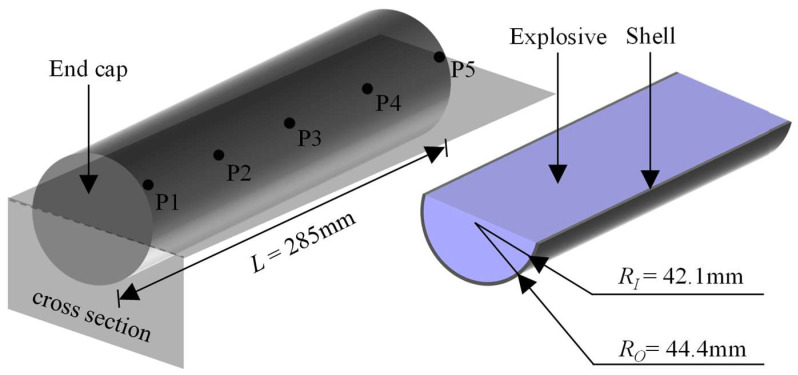
Schematic diagram of the validating model of the shelled charge.

Based on the shell size and charge type in the explosion test, the numerical model is shown in Figure 8. It utilizes a tetrahedral grid of 2 mm size, with a total grid number of 3.6 × 10^5^. The test charge is represented by a blue cylinder with radius *R_I_* = 42.1 mm, while the grey cylindrical shell with radius *R_O_* = 44.4 mm and wall thickness *δ* = 2.3 mm symbolizes the metal shell. These two components make uniform contact, with the length of the shell being *L* = 285 mm.

Five evenly spaced monitoring markers are positioned on the shell’s surface to capture the information of fragments displacement and velocity at the designated location. The layout of these points is shown in Figure 8. Specifically, point P3 serves as the central point of the shell busbar, while points P1 and P2 are symmetric to points P5 and P4, respectively, relative to the radial center section of the shell.

In order to accurately simulate the fracture process of the shell, the bilinear fracture model is used to describe the fracture evolution process between metal elements. First, the elastic contact force is calculated by Equation (9), where *F_n_* and *F_s_* denote the normal and tangential contact forces, respectively, *K_n_* and *K_s_* denote the normal and tangential contact stiffness, respectively, and Δ*d_n_* and Δ*d_s_* denote the relative displacement increments in the normal and tangential directions, respectively.

(9) $$\{\begin{array}{l} F_{n}(t+\Delta t)=F_{n}(t)-K_{n}\times \Delta d_{n}\\ F_{s}(t+\Delta t)=F_{n}(t)-K_{s}\times \Delta d_{s} \end{array}$$

**Figure 9:**
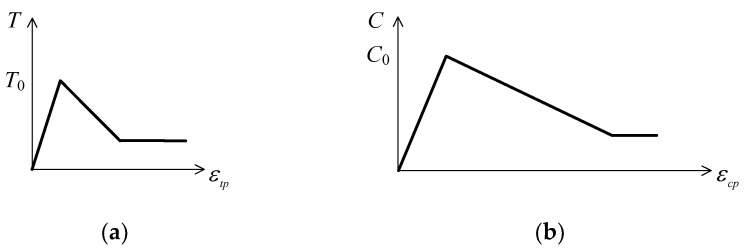
Schematic diagram of structural surface softening model: (**a**) tensile failure and (**b**) shear failure.

In order to calculate the progressive failure process of the material, the Mohr-Coulomb criterion and the maximum tensile stress criterion are introduced as follows:

(10) $$\{\begin{array}{l} \begin{array}{l} (1)\;\mathrm{tensile}\;\mathrm{failure}\\ \begin{array}{l} if\;\;\;\;-F_{n}(t+\Delta t)\geq T(t)\times A_{c}\\ then\;\;F_{n}(t+\Delta t)=-T(t)\times A_{c},\;T(t+\Delta t)=0 \end{array} \end{array}\\ \begin{array}{l} (2)\;\mathrm{shear}\;\mathrm{failure}\\ \begin{array}{l} if\;\;\;\;\;F_{s}(t+\Delta t)\geq F_{n}(t+\Delta t)\times \tan f+C(t)\times A_{c}\\ then\;F_{s}(t+\Delta t)=F_{n}(t+\Delta t)\times \tan f+C(t)\times A_{c},C(t+\Delta t)=0 \end{array} \end{array} \end{array}$$

where *T* denotes the tensile strength of the current time step, *C* denotes the cohesive force of the current time step, *f* denotes the internal friction angle, and *A_c_* denotes the contact area. The structural softening model of tensile failure and shear failure is shown in Figure 9.

In order to characterize the effect of the strain rate on the mechanical parameters of the metal, the relationship between the cohesion and tensile strength of the interface and the tangential strain rate and normal strain rate of the interface is introduced as follows in CDEM.

(11) $$\{\begin{array}{l} C=C_{0}[1+\alpha \ln(1+\dot{\gamma })]\\ T=T_{0}[1+\alpha \ln(1+\dot{\varepsilon })] \end{array}$$

where *C*_0_ and *T*_0_ denote the static cohesion and tensile strength, respectively, *γ* and *ε* denote the tangential and normal strain rates on the interface, respectively, and *α* denotes the strain rate coefficient.

The mechanical parameters of the Q235 steel used in the numerical simulation are shown in Table 3.

### 3.3. Verification of Numerical Results

The numerical simulation spanned a duration from 0 to 100 μs. The mass–quantity distribution of the natural fragments was counted. The resulting displacement and velocity characteristics of the fragments were recorded by marker points on the surface of the shell and the fracture process and damage/fracture type of the shell were recorded by cell monitoring.

#### 3.3.1. Initial Velocity of Fragments

**Figure 10:**
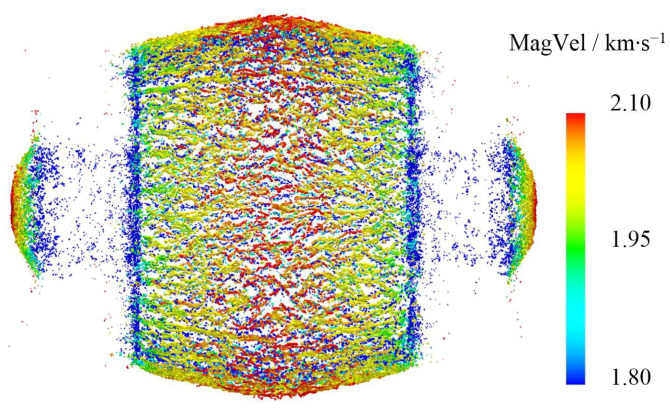
Velocity cloud diagram of fragments at 100 μs.

The velocity cloud diagram of fragments at 100 μs is shown in Figure 10. The resulting velocity distribution of the majority of these fragments falls within the range of 1.8–2.1 km/s. The maximum, average, and minimum value of the resulting velocity are *V_Max_* = 2.118 km/s, *V_average_* = 2.019 km/s, and *V_Min_* = 1.902 km/s, respectively.

Based on the data of the velocimetric target in Table 2, the initial velocity of the fragment was calculated as *V*_0_ = 2.22 km/s. The errors of *V_Max_*, *V_average_*, and *V_Min_* were 4.59%, 9.04%, and 14.32%, respectively. These numerical simulation results can effectively reflect the real situation of fragment scattering velocity in the test.

#### 3.3.2. Mass–Quantity Distribution

Existing fragment recovery technology faces significant limitations, making comprehensive recovery of all fragments following shelled charge explosion a challenging task. The Mott distribution theory has been extensively applied to assess the mass–quantity distribution of natural fragments. Furthermore, preceding research findings have corroborated the accuracy of the Mott distribution. This paper aims to examine the accuracy of numerical simulations, which is achieved by undertaking a comparative analysis between the numerical simulation outcomes and the established Mott theory.

**Figure 11:**
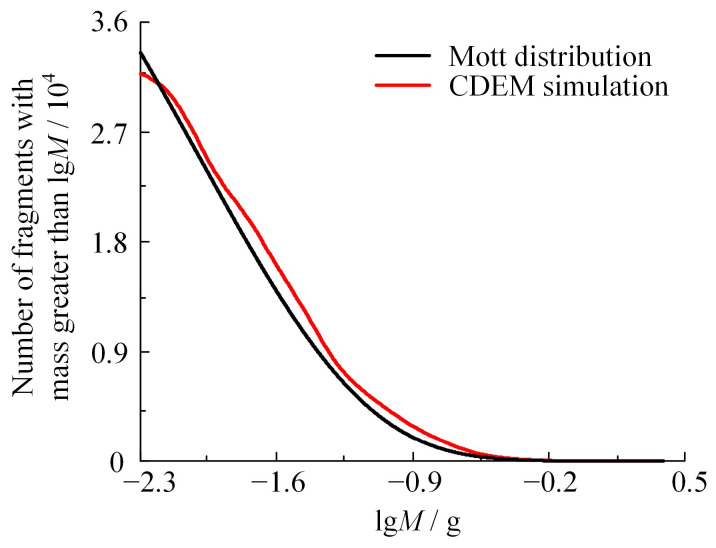
Mass–quantity distribution of fragments.

The shell examined in this study represents a quintessential thin-walled structure. It manifests fractures in two dimensions, subsequently leading to the formation of natural fragments. The mass–quantity distribution of the fragments is introduced as follows:

(12) $$N(m_{f})=\frac{M_{s}}{2\mu }\cdot e^{-{\left(\frac{m_{f}}{\mu }\right)}^{\frac{1}{2}}}$$

where *N*(*m_f_*) denotes the number of fragments with a mass greater than *m_f_* and 2*μ* denotes the average mass of broken fragments. Incorporating the principles of Mott Theory, Gurney and Sarmousakis proposed a *µ* formulation applicable for thin-walled cylindrical shells is introduced as follows:

(13) $$\mu^{1/2}=K\frac{\delta \left(D_{I}+\delta \right)}{D_{I}}\sqrt{1+0.5\left(\frac{M_{E}}{M_{S}}\right)}$$

where *K* denotes the parameter related to the performance of the explosive in g^1/2^/cm^7/6^. Concerning the known empirical parameters of monomeric explosives and the ratio of the components used in this test, the value of *K* was taken as 0.293 and *μ* = 0.01. The resulting fragment mass–quantity distributions of the Mott theoretical calculation and the numerical simulation are shown in Figure 11.

The fragments were divided into five weight categories for analysis, which were formed by considering the ratio of fragment count and total weight to that in various mass intervals. For each weight category, the counts of fragments computed by Mott’s theoretical calculation and derived from numerical simulations were compared. The discrepancy between these values is presented in Table 4:

As can be seen from Table 4, the error in four of these intervals is less than 20% when the simulation results are compared with those of the Mott theory. The interval between 0.01–0.0105 g is an exception, with a maximum error of 30.86%, attributable to the constraints of the smallest grid dimension in the numerical model. The minimum detectable fragment mass is 0.0074 g; hence, predictions for a total number of fragments closer to this mass might exhibit inaccuracies. Mott theory and numerical simulation estimate the total number of fragments as 3.486 × 10^4^ and 3.2827 × 10^4^, respectively, yielding a simulation-error margin of 5.83%. These numerical simulation outputs serve as appropriate reflections of the mass–quantity distribution of natural fragments.

Subsequent to this analysis, the implosion-induced fracture and fragmentation process of thin-walled shells can be accurately portrayed using continuum–discontinuum element method.

## 4. Discussion

The detonation of a shelled charge progresses through three phases: initial shell deformation and fracture, acceleration of fragments driven by detonation products, and a deceleration phase attributable to air resistance. Both the initial deformation/fracture and the fragment acceleration stages transpire within a very brief period, on the order of microseconds, while the deceleration stage progresses over a longer time scale, on the order of milliseconds. The fracture and fragmentation process of the shell were analyzed based on the verified numerical simulation results in the CDEM program.

### 4.1. Fracture Mechanism of the Thin-Walled Cylindrical Shell

Under the load of implosion, the shell expands, deforms, and breaks as pressure from the detonation products diminishes. The failure modes include both tensile and shear failure. Using the continuum–discontinuum element method, the virtual interface between cells slips and breaks under the influence of the detonation products. To investigate the mechanism of the shell’s fracture evolution, the damage to the spring and the fracture area of the shell element under shear and tensile loads were quantified based on the results of numerical simulations. The spring area of shear damage/fracture is on the order of 10^−1^ m^2^, while the spring area of tensile damage/fracture is on the order of 10^−4^ m^2^. The difference between the two is 1000 times, indicating that the failure of thin-walled shells is dominated by shear failure. At 100 μs, the shear damage area is 0.59 m^2^, while the shear fracture area is 0.38 m^2^; 64.41% of the blocks with shear damage evolve into shear fractures. The tensile damage area is 3.53 × 10^−4^ m^2^, while the tensile fracture area is 3.28 × 10^−4^ m^2^. The blocks with tensile damage evolve into tensile fractures with a ratio of 92.91%.

**Figure 12:**
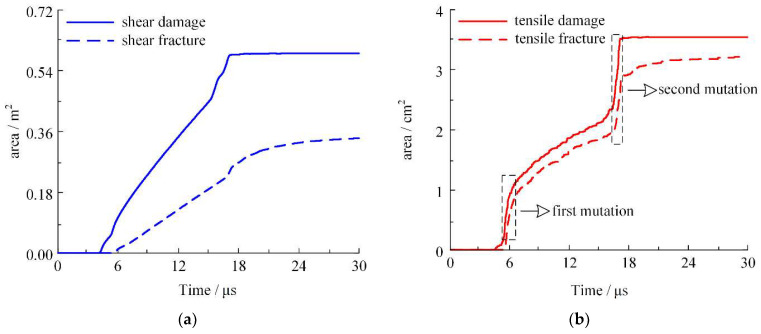
Damage/fracture spring area of the element under tensile/shear forces: (**a**) shear damage/fracture area of spring and (**b**) tensile damage/fracture area of spring.

At 30 μs, the shear and tensile spring damage and fracture area account for 99.97%, 90.22%, 99.97%, and 97.44% of the time of 100 μs, respectively, indicating that the fracture of the shell mainly occurs in the time interval of 0–30 μs. Therefore, the shell fracture evolution study was carried out around this time interval. The shell fracture evolution process is plotted in Figure 12.

Shear damage initially manifests in the shell at 4 μs, while tensile damage becomes evident at 4.5 μs, resulting in a 0.5 μs temporal discrepancy, with shear damage being the predecessor. Both shear and tensile fractures initiate almost simultaneously, at 5.3 μs and 5.4 μs, respectively. According to the law of propagation of stress waves in solids and the tensile stress guidelines for shell fracture, cracks begin to appear when the stress system of the shell wall reaches a critical state. Radial cracks appear in the circumferential tensile stress area of the shell and propagate along the tensile stress area. Furthermore, they are unable to extend into the compressive stress area, resulting in crack formation on the shell’s outer surface with subsequent propagation towards the inner surface. When the thickness of the compressive stress region existing in the inner wall of the shell is reduced to zero, the compressive stress of the inner wall of the shell is equal to the yield stress of the material. The crack propagates to the inner surface and the shell is completely broken. During this process, shear fractures occur in the direction of maximal shear stress when an exterior shell crack develops. The tensile fracture occurs perpendicularly to the principal stress and only after the crack extends to the inner surface. Consequently, due to the shell wall’s thickness, the tensile failure occurs later than the shear failure. This temporal disparity constitutes the tensile failure’s “hysteresis effect”.

The shell mainly incurs block damage at 4–18 μs. The shear damage area is 0.588 m^2^ and the tensile damage area is 3.52 × 10^−4^ m^2^ at 18 μs. At 18–30 μs, the shear damage area increases by 0.575% and the tensile damage area increases by 0.387%, indicating that the block damage area stops growing after the shell has completely broken. Most of the block fracture occurs simultaneously with block damage, though it continues to grow after 18 μs. In the three stages of 4–18 μs, 18–30 μs, and 30–100 μs, the area produced by shear fracture accounted for 71.046%, 21.895%, and 7.059% of the total value, respectively. Additionally, the area produced by tensile fracture accounted for 88.644%, 9.759%, and 1.597% of the total value, respectively. This indicates that after 18 μs the shear fracture area increased slowly until 100 μs, while the tensile fracture area ended before 30 μs.

**Figure 13:**
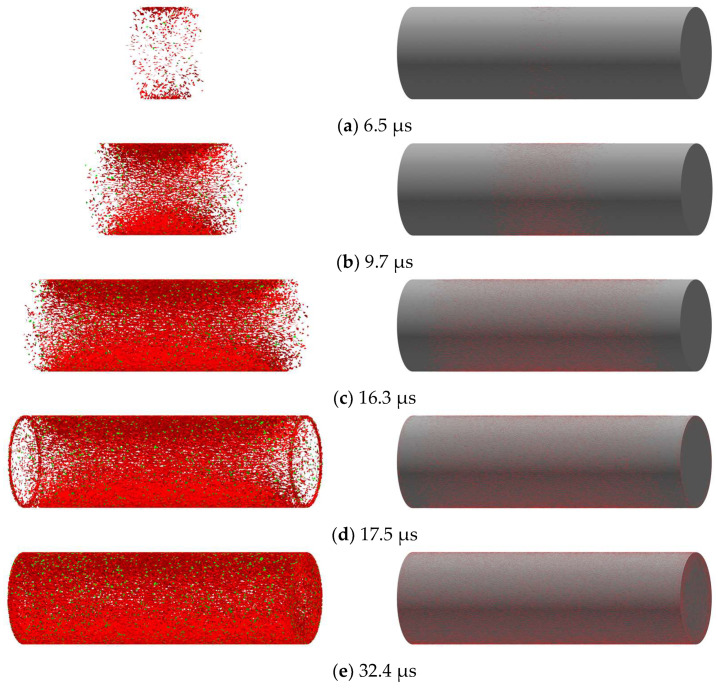
Shell failure evolutionary process.

The crack proliferation cloud map of the shell is shown in Figure 13. For convenience of observation, the ratio distance of the cloud map is set to 0. Red and green denote the shear and tensile failure, respectively. The left side shows the cloud map depicting the failure face piece, while the right side shows the cloud map illustrating the presence of surface cracks on the shell. As can be seen from the cloud map, the shell crack propagates along the axial direction to both ends. Detonation products reach the end cover of the shell at 16.3 μs, completing more than 99.5% of the damage area growth. At 32.4 μs, the shear fracture area of 92.94% and tensile fracture area of 98.41% are increased, with a shell expansion for the initial diameter of 1.8 times.

As seen in the selected part of the dashed line frame in Figure 12b, the growth of the tensile damage/fracture area undergoes two mutations, with the mutation occurrence intervals for damage and fracture overlapping. The first mutation occurs in the time range of 5.5–6.5 μs, corresponding to the failure cloud map in Figure 13a. At this stage, the detonation products swiftly reach the shell wall, initiating the formation of a crack along the circumference starting from the center of the shell. During this stage, the evolution time represents only 1% of the total time, while the growth area attributed to tensile damage accounts for 18.70% of the total area and the growth area associated with tensile fracture amounts to 24.91% of the total area. The second mutation occurs in the time range of 16.3–17.1 μs, corresponding to the failure cloud map Figure 13c,d. Detonation products reach the end cover of the shell earliest at this stage. The crack is generated and expands inward along the outermost ring on the end cover. During this stage, the evolution time represents a mere 0.8% of the total time, while the growth area attributed to tensile damage accounts for 32.77% of the total area and the growth area associated with tensile fracture amounts to 18.71% of the total area. The two mutations account for 1.8% of the total time, while the damage and fracture areas account for 51.47% and 43.62% of the total area, respectively, indicating that the tensile failure of the shell mainly occurs in the middle and ends.

**Figure 14:**
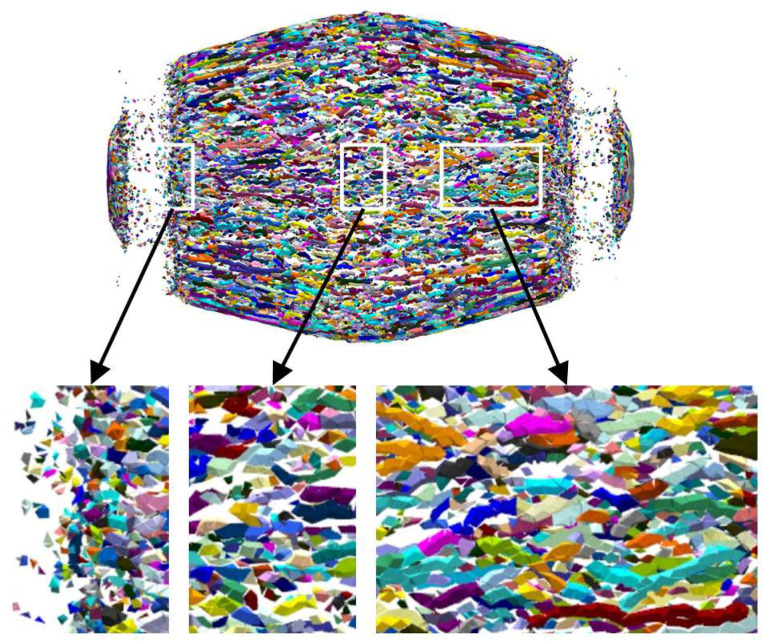
Fractal cloud diagram of fragments.

To analyze the discrepancies between naturally created fragments due to shear and tensile failure, the fractal of fragments at 60 μs is shown in Figure 14. At this specific duration the fracture of the shell is completed, leading to the enhanced visibility of fragments as they disperse uniformly. The detailed local magnification reveals three distinct regions. The two regions of the side wall centre and the end cover, correlating to the two mutations of tensile failure, exhibit lesser fragments volumes and fewer bar-shaped fragments relative to other areas. This suggests that tensile damage significantly influences the fragmentation outcome in both regions.

### 4.2. Scattering Characteristics of the Natural Fragments

**Figure 15:**
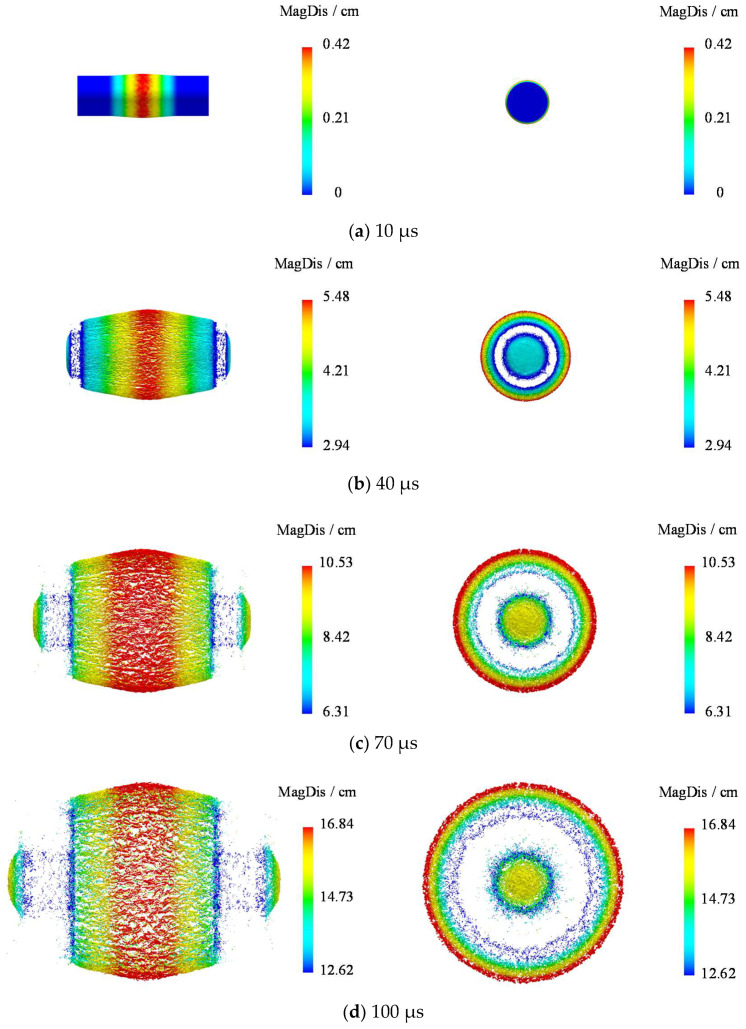
Scattering process of fragments.

The displacement cloud diagram resulting from natural fragments at various moments is shown in Figure 15. This diagram represents fragments from both the side wall and end perspectives, as identified on the left and right sides, respectively. Notably, the displacement recorded at 100 μs is chiefly within the range of 12.62–16.84 cm, by which time the acceleration of the fragments has stopped.

To investigate the displacement characteristics of fragments and expansion characteristics of the shell at different acceleration stages, the dimensionless parameter *β* = MagDis/*R_O_* is introduced to represent the relationships between the resulting displacement of the fragments and the shell radius. When calculating *β* at a certain moment, the average displacement value of all fragments at that moment is taken as MagDis. The relative specific volume can be considered as $\bar{v}$ = (*β* + 1)^3^. At $\bar{v}$ = 7 and $\bar{v}$ = 13; the value of *β* is 0.913 and 1.351, corresponding to 36.412 μs, 47.852 μs, respectively.

**Figure 16:**
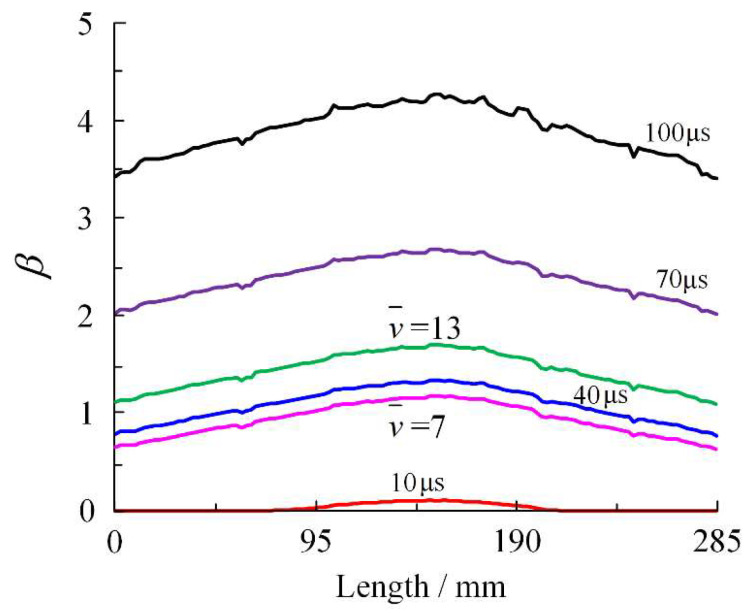
Resulting displacement–time curve of fragments.

The resulting displacement distribution curves of fragments along the shell busbar are shown in Figure 16. These curves exhibit a smooth trend during the early scattering phase at 10 μs and 40 μs, transitioning to substantial fluctuations during the later scattering phase at 70 μs and 100 μs. This suggests that the dispersion of fragment displacement increases as the shell fractures and the fragments separate. At the corresponding moments of $\bar{v}$ = 7 and $\bar{v}$ = 13, the average resultant velocity of fragments is 3.844 cm and 5.688 cm, respectively. Utilizing the methodology presented in this paper improves the representation ability of fragment displacement by 47.97% compared to the cylinder test.

**Figure 17:**
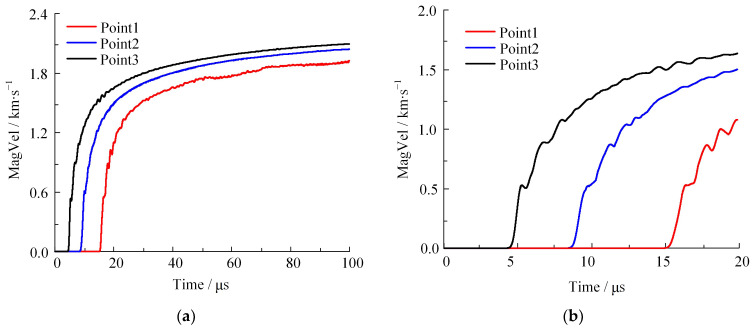
Velocity–time history curve of fragments in acceleration stage: (**a**) full acceleration curve and (**b**) starting moment of acceleration.

The fragments generated by shell fracture are accelerated by detonation products. Through the five marked points in Figure 8, the resulting velocity and displacement evolution of the location are recorded. Considering the symmetry between points P1 and P5 as well as that between P2 and P4, the evolution of the respective fragment parameters shares a convergent pattern. The fragments at P1, P2, and P3 are correspondingly labeled as Point1, Point2, and Point3. Figure 17 showcases the acceleration time history curves for these three fragments.

Figure 17a shows the full curve of fragment acceleration. The fragments Point1, Point2, and Point3 are accelerated, with a final resultant velocity of 1.916 km/s, 2.034 km/s, and 2.086 km/s, respectively. Additionally, they attain 80% of their final velocities at 21 μs, 26 μs, and 32 μs. At the corresponding moments of $\bar{v}$ = 7 and $\bar{v}$ = 13, the average velocity of the fragments is 1.754 km/s and 1.847 km/s, respectively. The two values are 86.88% and 91.46% of the velocity at 100 μs, respectively. Utilizing the methodology presented in this paper improves the representation ability of fragment velocity by 5.30% compared to the cylinder test. Based on the analysis of displacement and velocity, the JWL equation of state can describe the scattering process of fragments over a wider range by calibrating it with DLCHEQ.

**Figure 18:**
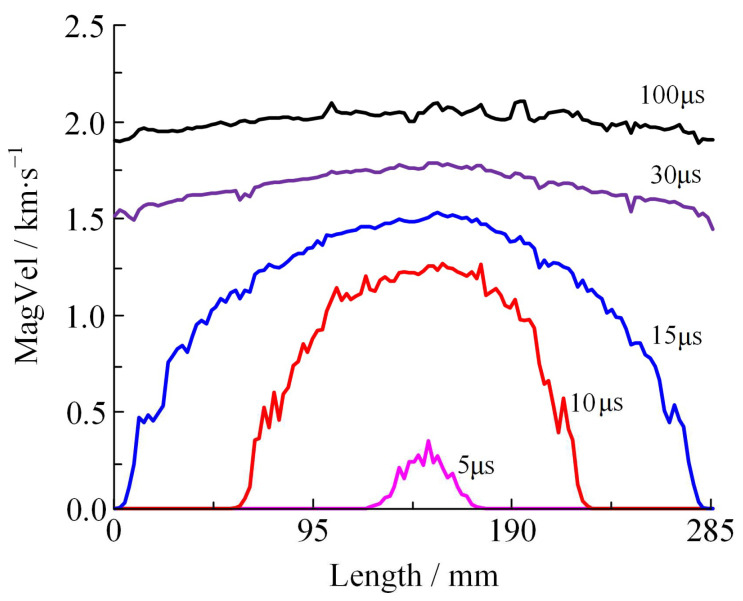
Velocity–time curve of fragments.

Figure 17b shows the initial curve of fragment acceleration. The earliest moment of fragment acceleration is at 4 μs and the latest is at 15 μs, which corresponds to the beginning of the fracture of the shell and the moment when the detonation products reach the end cover. This result shows that fragments develop from the centre of the busbar to both ends. In order to explore the acceleration law of global fragment scattering of the shell, the resulting fragment velocity distribution curves along the shell busbar at 5 μs, 10 μs, 15 μs, 30 μs, and 100 μs are plotted in Figure 18.

The velocity distribution of the resulting fragments can be divided into three stages. During stage A (0–18 μs) the shell undergoes partial fragmentation, experiencing acceleration from the center to both ends under the expansive force exerted by the detonation products. The resultant velocity of fragments at different positions is different. For example, at 15 μs the maximum velocity of the fragment reaches 1.529 km/s, while the end of fragment has a velocity of 0. During stage B (15–30 μs), detonation products are continuously loaded for the second time after the shell has completely broken. At 30 μs the maximum velocity of the fragment is 1.787 km/s, while its minimum velocity is 1.447 km/s. The average velocity of all fragments is 1.665 km/s, with 0.34 km/s separating the highest and lowest values, indicating that the velocity of fragments at different positions is close. During stage C (30–100 μs) there is a substantial dissipation of detonation products, leading to a decrease in the propelling force exerted by the fragments. At 100 μs the average resultant velocity of all fragments is 2.019 km/s, i.e., the average velocity of the fragments increases by 0.354 km/s in the time interval of 70 μs.

Stages A and B collectively constitute 30% of the total acceleration time, with the average velocity at 30 μs reaching 88.51% of the final average velocity achieved upon completion of the acceleration process. These stages serve as the primary acceleration stages for the fragments.

## 5. Conclusions

This study analyzes the fracture and fragmentation of a thin-walled cylindrical shell under implosion force, and presents a strategy to gauge an explosive’s work capability. The conclusions obtained are as follows:

1. Using the thermochemical equation of state code DLCHEQ, the isentropic expansion curve of detonation products in the range of <13 was computed. Subsequently, the JWL EOS coefficients were calibrated across a wider range of temperature and pressure through curve fitting. This approach can successfully delineate an explosive’s work capacity, as the derived equation of state accurately reflects the cylinder test’s measurement results. Through this method, the fragmentation process of the shell can be described over a wider range.
2. Simulation of dynamic fracture process of the shell was undertaken based on the continuum–discontinuum element method. The validity of the numerical simulation results was corroborated through the Mott mass–quantity distribution theory and shelled charge explosion test velocity measurement. The discrepancy between the average initial velocity of fragments and the experimental value was 9.04%. The mass–quantity distribution curve of the fragments aligned well with the theoretical curve, demonstrating a discrepancy of only 5.83% in the total fragment count when compared to Mott’s theoretical prediction. From these results, it can be seen that the scattering process of natural fragments generated by the thin-walled shell under implosion load is accurately described by the method.
3. The thin-walled shell employed in this study primarily failed through shearing, generating strip fragments. In the evolution of tensile failure, two mutations generate local particle fragments, which constitute 1.8% of the total time and cover over 50% of the total failure area. Compared to shear failure, there is a “hysteresis effect” at the beginning of tensile failure and the hysteresis period is 0.5 μs. The shell’s failure process mainly lasts for 4–18 μs, after which the unit’s damage area stops growing and the fracture area begins to expand slowly. According to the processes of shell fracture and fragmentation, the acceleration process of natural fragments can be divided into three stages. The primary stages of fragment generation and acceleration are stages A and B, respectively. At 30 μs the shear/tensile damage/fracture area accounts for over 90% of the final value, and the average resultant velocity of fragments is 88.51% of the final value. The velocity and shear fracture area continue to gradually rise in stage C, indicating that fragments continue to be accelerated by detonation products outside the calibration range of the cylinder test. The final resultant velocity of fragments ranging from 1.8 to 2.1 km/s at 100 μs.

## Figures and Tables

**Table 1:** Coefficients of the JWL EOS from calibration.

*A*	*B*	*C*	*R_1_*	*R_2_*	*ω*
8	0.16	0.004	4.3	1.1	0.36

**Table 2:** Velocimetric target data at 4 m and 5.2 m.

		Velocity/km·s^−1^			
		1	2	3	Average
Test distance/m	4	1.49	2.00	1.92	1.80
	5.2	2.03	1.60	1.43	1.69

**Table 3:** Coefficients of the JWL EOS from calibration.

Density *ρ*/g·cm^−3^	Elastic Modulus *E*/GPa	Poisson Ratio	Cohesion Force *C*_0_/MPa	Tensile Strength *T*_0_/MPa	Internal Friction Angle *f*/°
7.85	198	0.33	15	500	40

**Table 4:** Distribution of fragments number at different quality intervals.

		Quality Interval/g				
		0.01–0.0105	0.0105–0.0205	0.0205–0.0405	0.0405–0.0705	>0.0705
Mott theory	Number	9188	8101	7157	4308	4779
	Proportion of total quantity	27.4%	24.16%	21.34%	12.85%	14.25%
	Proportion of total mass	1.23%	19.81%	24.23%	19.81%	34.92%
CDEM	Number	6353	6959	7852	4867	5732
	Proportion of total quantity	20.00%	21.91%	24.72%	15.32%	18.05%
	Proportion of total mass	3.31%	6.59%	14.75%	16.20%	59.15%
Error of fragments quantity		30.86%	14.10%	9.71%	12.98%	19.44%

## Data Availability

The data presented in this study are available on request from the corresponding author.
